# Genome-wide identification and expression analysis disclose the pivotal *PHOSPHATIDYLETHANOLAMINE BINDING PROTEIN* members that may be utilized for yield improvement of *Chenopodium quinoa*

**DOI:** 10.3389/fpls.2022.1119049

**Published:** 2023-01-10

**Authors:** Qi Wu, Xue Bai, Mengping Nie, Li Li, Yiming Luo, Yu Fan, Changying Liu, Xueling Ye, Liang Zou

**Affiliations:** ^1^ Key Laboratory of Coarse Cereal Processing, Ministry of Agriculture and Rural Affairs, Chengdu University, Chengdu, Sichuan, China; ^2^ Sichuan Engineering & Technology Research Center of Coarse Cereal Industralization, Chengdu University, Chengdu, Sichuan, China; ^3^ School of Food and Biological Engineering, Chengdu University, Chengdu, Sichuan, China

**Keywords:** FT, TFL, MFT, flowering, yield, Chenopodium quinoa

## Abstract

Quinoa (*Chenopodium quinoa*) is a prospective orphan crop that needs yield improvement. Previous studies indicate *PHOSPHATIDYLETHANOLAMINE BINDING PROTEIN* (*PEBP*) family genes are highly associated with the key agronomic traits of crops. Characterizing the pivotal *PEBP* genes will speed up the domestication and yield improvement of quinoa. Previous investigations on *PEBP* genes of *Chenopodium* species indicated that, the *PEBP* genes, despite in the same subclade, may have experienced functional diversification. Especially, the allotetraploidy (AABB) and numerous segmental duplications and chromosomal rearrangements in quinoa make it more difficult to understand the functions of *PEBP* genes. More recently, 6 quinoa *FT* subfamily genes were predicted to be related to flowering of quinoa. However, investigation on the whole *PEBP* family members is still lacking. In this study, we obtained 23 *PEBP* genes, including 5 *MFT*, 11 *FTL* and 7 *TFL* genes. We found 7 orthologous gene pairs, from sub-genome A and sub-genome B, respectively, showing collinearities with sugar beet. Evolution analysis on *PEBP* genes of two quinoa sub-genomes, sugar beet and relatives of diploid ancestors indicated that, the reasons for gene duplication events varied and 4 tandem duplications are the major reason for *PEBP* family expansion. Tissue-specific expression analysis suggested that expression patterns are mostly differing between orthologous gene pairs. Analysis on gene expressions at 6 stages suggested the possible positive roles of *CqFTL1*/*CqFTL2*, *CqFTL5*, *CqFTL8*, *CqFTL6*/*CqFTL9* and *CqTFL6*/*CqTFL7*, and negative roles of *CqTFL1*/*CqTFL2*/*CqTFL3*, *CqTFL4*/*CqTFL5* in inflorescence branching. Expression analysis in ABA-treated seed, in combination with the *cis*-acting element distribution analysis, indicated that *CqMFT2*, *CqMFT3* and *CqMFT4* may regulate seed germination *via* ABA signaling. Observations on responses to night break and photoperiod changes highlighted the roles of *CqFTL5* and *CqFTL8* under short day, and *CqFTL6* under long day for quinoa flowering. Further, co-expression network analysis indicated that 64 transcription factors may act upstream of *CqFTL5* and *CqFTL8* to regulate flowering. Together, this study will help us identify the pivotal *PEBP* genes that may be utilized for quinoa breeding in future.

## Background

Quinoa (*Chenopodium quinoa*) is a prospective orphan crop due to the nutritional components in its seed and high tolerance to various abiotic stresses that could ensure its growth in marginal lands (Lopez-Marques et al., 2020). Nowadays, because of the increasing global demands for quinoa, yield improvement per unit area and expansion of cultivation area should be achieved. As demonstrated in many studies, flowering time regulation not only is tightly associated with inflorescence morphology and yield, but also is related to the adaption in higher latitudes (Song et al., 2015). Thus, identifying the genes highly associated with flowering and inflorescence morphology is essential for fast domestication and yield improvement of quinoa.

Transition from vegetive to reproductive stage is influenced by both internal and environmental cues (Song et al., 2015). Photoperiod is the major pathway regulating floral transition. In favorable season, day length signal is transmitted to the major floral integrator *FLOWERING LOCUS T* (*FT*) (Taoka et al., 2013; Tsuji et al., 2013; Holt et al., 2014; Kyozuka et al., 2014; Song et al., 2015). *FT* encodes for florigen protein that moves through the phloem from leaves to the shoot apical meristem (SAM), where partners with bZIP transcription factor FD and 14-3-3 proteins to form floral activating complex (FAC), which in turn promotes expressions of the floral identity genes *APETALA1* (*AP1*) and *LEAFY* (*LFY*) (Taoka et al., 2013; Tsuji et al., 2013; Holt et al., 2014; Kyozuka et al., 2014; Song et al., 2015). *FT* belongs to *PHOSPHATIDYLETHANOLAMINE BINDING PROTEIN* (*PEBP*) family. *PEBP* family contains three subfamilies, namely *FT*, *TERMINAL FLOWER 1* (*TFL1*) and *MOTHER OF FT AND TFL1* (*MFT*), all of which exert important roles in plant growth and development (Karlgren et al., 2011; Wickland and Hanzawa, 2015). In contrast to the floral-inducing role of *FT*, *TFL1* functions as a floral repressor and maintains vegetative growth by repressing *AP1* and *LFY* (Hanano and Goto, 2011; Kaneko-Suzuki et al., 2018; Goretti et al., 2020). The spatio-temporal expression of *FT*/*TFL1* affects flowering time, inflorescence architecture and final yield.

Up to date, a good many evidences have demonstrated the important roles of *FT*-, *TFL1*- and *MFT*-*like* genes in agronomic traits regulation. Overexpression of the rice (*Oryza saltiva*) *TFL1* homologs, *RICE CENTRORADIALIS-like 1/2* (*RCN1/2*), led to delayed heading and generation of higher-order panicle branches (Nakagawa et al., 2002), whereas RNA interfering (RNAi) of rice *TFL1* resulted in advanced heading and reduced branches (Liu et al., 2013). The maize (*Zea mays*) plants ectopic expressing *ZEA CENTRORADIALIS 2*/*4*/*5* (*ZCN2*/*4*/*5*) produced increased lateral branches (Danilevskaya et al., 2010). The wheat (*Triticum aestivum*) plants overexpressing *TaTFL1-2D* generated increased spikelets (Wang et al., 2017). In the background of mutant *self-pruning* (*sp*, homolog of *TFL1*), the tomato (*Solanum lycopersicum*) plants with heterozygous *single flower truss* (*sft*/+) (*sft*, homolog of *FT*) produced heterosis and dramatically increased number of fruits (Krieger et al., 2010; Jiang et al., 2013). A natural variant of the *TFL1* homolog *CENTRORADIALIS* (*HvCEN*) contributed to the spring growth habit of cultivated barley (*Hordeum vulgare*) (Comadran et al., 2012). *HvCEN* interacts with *HvFT3* to control spikelet initiation and grain number of barley (Mulki et al., 2018; Bi et al., 2019). In addition to the classical florigen function, the rice *FT* homolog *HEADING DATE 3A* (*HD3A*) also regulates shoot branching (Tamaki et al., 2007; Tsuji et al., 2015). In potato (*Solanum tuberosum*), the *FT* homolog *StSP6A* is required for tuberization transition (Navarro et al., 2011). *MFT*, ancestral gene of *FT* and *TFL1*, is a key regulator in seed germination. *OsMFT2* negatively regulates seed germination *via* ABA pathway and the knock-out mutant exhibited pre-harvest sprouting phenotype (Song et al., 2020). The wheat *MFT* was revealed to be tightly linked with the seed dormancy QTL *QPhs.ocs-3A.1* (Nakamura et al., 2011). The conserved functions of *PEBP* homologs were found among different plant species. However, due to the differing expression patterns and multiple copies of *PEBP* family, the *PEBP* genes, even in the same subfamily, may have distinct functions. For example, in soybean (*Glycine max*), *FT5a* is involved in post-flowering stem growth other than the flowering-inducing role shared with *FT2a* (Takeshima et al., 2019). In rice, the *FT* homolog *HD3A* is the principal flowering regulator under short day, while the other *FT* homolog *RICE FLOWERING LOCUS T1* (*RFT1*) mainly functions under long day (Komiya et al., 2008; Komiya et al., 2009).

As the importance of *PEBP*s in yield regulation has been demonstrated in many crops, characterizing and manipulating of the significant *PEBP* genes in quinoa will help improve the yield and cultivation area of quinoa. Recently, a few studies on *FT* subclade genes were carried out in Amaranthaceae family. In sugar beet (*Beta vulgaris*), two *FT* paralogs, *BvFT1* and *BvFT2*, were identified. However, they harbor antagonistic functions in flowering (Pin et al., 2010). In *C. rubrum*, two *FT* homologs, *CrFTL1* and *CrFTL2*, were identified, in which only *CrFTL1*, rather than *CrFTL2*, was up-regulated during floral transition (Chab et al., 2008). In *Chenopodium* species, the expression of *FTL1* was correlated with floral induction in *C. suecicum* and short-day type *C. ficifolium*, whereas was not correlated with that in long-day type *C. ficifolium* (Storchova et al., 2019). These results suggested that those *PEBP*s of *Chenopodium* species, despite in the same clade, may have experienced functional diversification. Quinoa, an allotetraploid (AABB), had experienced numerous chromosomal rearrangements and chromosome fusions (Jarvis et al., 2017), which may increase the functional complexity of *PEBP* family. More recently, the quinoa *FT* subfamily genes were identified and their evolutionary relationships with other plants were assessed (Štorchová, 2020). Expressions of six *CqFT* genes were compared in early- and late-flowering quinoa accessions (Patiranage et al., 2021). Haplotypes of two *CqFT* genes were predicted to be associated with the photoperiod sensitivity of quinoa (Patiranage et al., 2021). These studies have improved our understanding of the plausible functions of quinoa *FT-like* subfamily members. Yet, functions of quinoa *TFL1-like* and *MFT-like* subfamily genes remain mysterious. Complete investigations into gene duplication, gene structure, *cis*-acting element in the promotor, and more important, the transcriptional changes of the whole *PEBP* family in various progresses are still required to further elucidate their specific roles. In this study, we analyzed the phylogenetic relationships, collinearities and duplication events between *PEBP* genes of sub-genome A and sub-genome B and relatives of diploid ancestors, and assessed their expression changes during inflorescence development and seed germination and detected their responses to night break and photoperiods, and further performed co-expression network analysis to predict the transcription factors upstream of *PEBP* genes. The results of this study will help identify the pivotal *PEBP* genes governing flowering time, inflorescence branching and seed germination, which may be utilized for quinoa breeding in future.

## Materials and methods

### Identification, and phylogenetic analysis of PEBP homologs from different plants

To identify the PEBP genes in various plant species, we performed BLASTP (E-value<1.0e-15) search against genomes of *Arabidopsis thaliana*, *Spinacia oleracea*, *Oryza sativa* and *Beta vulgaris* in Phytozome V13 (https://phytozome-next.jgi.doe.gov), and *C. pallidicaule* and *C. suecicum* in *Chenopodium* database (https://www.cbrc.kaust.edu.sa/chenopodiumdb/), using FT (AT1G65480.1), TFL (AT5G03840.1) and MFT (AT1G18100.1) protein sequences of Arabidopsis as the queries. Then those homologs were aligned with the Hidden Markov Model (HMM) profile of PEBP domain (PF01161) using Pfam search (E-value<1.0e-10) (Finn et al., 2016) to ensure those homologs habor a PEBP domain. Multiple sequence alignment of PEBP sequences from various species was performed using CLASTALW (Thompson et al., 2002). Phylogenetic tree was constructed using MEGA 11.0 (Tamura et al., 2021) based on the Neighbor-Joining method (Dohm et al., 2013) with a bootstrap value of 1000.

### Chromosomal location, gene structure and conserved motif analysis

The General Feature Format (GFF) file and chromosome-scale genome sequence of quinoa were downloaded from Phytozome V13 database (https://phytozome-next.jgi.doe.gov/info/Cquinoa_v1_0). Based on these two files, the physical location on chromosomes, and intron and exon structures of PEBP genes were determined and visualized by using the two programs of Gene Location Visualize and Gene Structure View in TBtools (Chen et al., 2020). Multiple Em for Motif Elicitation (MEME) program (https://meme-suite.org/meme/tools/meme) was used to identify the conserved motifs in PEBP proteins setting the maximum motif count of 8. The motif analysis results were illustrated using the Gene Structure View program in TBtools (Chen et al., 2020).

### Collinearity and gene duplication events analysis between *PEBP* genes of different species

The GFF file and chromosome-scale genome sequence of sugar beet (*Beta vulgaris*) were downloaded from Phytozome V13 database (https://phytozome-next.jgi.doe.gov/info/Bvulgaris_EL10_1_0). The GFF files and chromosome-scale genomes of quinoa and sugar beet were input into the Multiple collinear scanning tool (MCScanX) in TBtools (Chen et al., 2020) and the collinearity between quinoa and sugar beet genomes was analyzed. Then the collinearities between *PEBP* genes from quinoa and sugar beet were determined. Meanwhile, the duplication events among quinoa *PEBP* genes were analyzed. The results were visualized by using the Advanced Circos program in TBtools (Chen et al., 2020).

### Distribution of *cis-*acting element in quinoa *PEBP* gene promoters

The 2000bp sequences upstream of the translation start site of *PEBP* genes were recognized as promoter regions. The promoter sequences were uploaded to the Plant *Cis*-Acting Regulatory Elements (PlantCARE) database to search *cis-*acting elements. The physical distribution of various *cis-*acting elements was displayed using Simple BioSequence Viewer in TBtools (Chen et al., 2020).

### Expression analysis of PEBPs in various tissues and biological events

For tissue-specific expression pattern analysis, we used the RNA-seq data from Zou et al. (Zou et al., 2017). Raw data of various quinoa tissues, including 1-week-old seedling, stem, leaf, inflorescence from 6-week-old plants and dry seed, was downloaded from Sequence Read Archive (https://www.ncbi.nlm.nih.gov/sra) of the BioProject PRJNA394651. The fragments per kilobase of transcript per million fragments mapped (FPKM) value of each gene was calculated with the previously described methods (Wu et al., 2019; Wu et al., 2020; Wu et al., 2021). For gene expression analysis in quinoa inflorescences at six developmental stages, we investigated the FPKM values of *PEBP* genes in our published transcriptome data (Wu et al., 2019). YP1, YP2, YP3 and YP4 represent the young non-branching panicles, whereas P1 and P2 stand for the panicles of elder branching stages. Raw data generated from six-stage inflorescence samples was downloaded from SRA (BioProject PRJNA511332), and was further processed with the bioinformatic pipeline methods described before (Wu et al., 2020). To know gene expression changes of *PEBP* genes during seed germination, we used the published transcriptome data of our laboratory (Wu et al., 2020). We compared the expression levels (FPKM value) of *PEBP* genes in control and Abscisic acid (ABA)-treated seeds 5h and 15h after imbibition. To investigate *PEBP* gene expression changes in response to night-break (NB) treatment, we used our published RNA-seq data generated from leaf samples treated by short-day and NB conditions (Wu et al., 2021). To investigate the *PEBP* gene diurnal expression pattern changes in response to photoperiod, two-week-old quinoa seedlings were cultivated under short-day (8h/16h, light/dark) and long-day (16h/8h, light/dark) conditions for sampling and RNA-seq analysis. The top two fully-expanded leaves of 3~5 plants were harvested at the time point of 17:00, 20:00, 23:00, 02:00, 05:00, 08:00, 11:00 and 14:00, with two biological replicates. All the leaf samples were subjected to RNA extraction, high-throughput sequencing and data analysis as previously described (Wu et al., 2019; Wu et al., 2021).

### Prediction of the co-expressed transcription factors with *PEBP*s

The expression profiles of 32 samples covering 16 time points of SD and LD were subjected to weighted gene co-expression network analysis (WGCNA) by applying R package, with the parameters set as following: gene expression threshold: FPKM≥1.0, power=1, minimum module size=30, minimum height for merging modules=0.25. As a result, 3972 genes were sorted into 6 co-expressed modules. *CqFTL5* and *CqFTL8* with were clustered into the blue module containing 934 co-expressed genes. Then all the 934 protein sequences in blue module were uploaded to PlantTFDB v5.0 website (Jin et al., 2017) (http://planttfdb.cbi.pku.edu.cn) for TF prediction.

## Results and discussion

### Identification, phylogenetic relationship and chromosomal location analysis of *PEBP*s in quinoa

By using BLASTP and Pfam search methods, 23 *PEBP* homologous genes were identified in quinoa genome. The shortest quinoa *PEBP* gene (AUR62033889, named *CqFTL3*), encoding for 88 amino acid residues (**Table 1**), was predicted to harbor an incomplete PEBP domain. The longest *PEBP* gene (AUR62013052, named *CqFTL2*), encoding for 339 amino acid residues (**Table 1**), was predicted to harbor two PEBP domains. These results are in line with the points in previous study (Jarvis et al., 2017; Štorchová, 2020), indicating that *CqFTL3* may be a pseudogene and *CqFTL2* may experience tandem duplication.

**Table 1:** Summary of *PEBP* gene family in *C. quinoa* and *B. vulgaris*.

Subfamily	Sub-genome	Gene ID	Gene name	Chr	Start-end position (+/- strand)	CDS (bp)	Protein (aa)	Ortholog in *B. vulgaris*	Ortholog ID	Orthologs in quinoa	Ratio (A: B)	Ratio (Cq: Bv)
**MFT**	A	AUR62029959	*CqMFT1*	Chr08	39662874-39666112 (+strand)	519	173				3:2	5:2
	A	AUR62012495	*CqMFT2*	Chr02	4599298-4601543 (-strand)	537	179					
	B	AUR62014699	*CqMFT3*	Chr01	29212627-29215543 (-strand)	537	179					
	B	AUR62014698	*CqMFT4*	Chr01	29210009-29211182 (-strand)	537	179	*BvMFT1*	EL10Ac8g20548.1	*CqMFT5*		
	A	AUR62012496	*CqMFT5*	Chr02	4594321-4597301 (-strand)	483	161	*BvMFT1*	EL10Ac8g20548.1	*CqMFT4*		
**FTL**	A	AUR62010060	*CqFTL1*	Chr15	4930835-4933952 (-strand)	540	180	*BvFT1*	EL10Ac9g21401.1	*CqFTL2*	5:6	11:3
	B	AUR62013052	*CqFTL2*	Chr17	79266951-79277600 (+strand)	1017	339	*BvFT1*	EL10Ac9g21401.1	*CqFTL1*		
	A	AUR62033889	*CqFTL3*	Chr10	48544986-48545464 (+strand)	264	88					
	A	AUR62000271	*CqFTL4*	Chr12	3192361-3196369 (+strand)	594	198	*BvFT2*	EL10Ac4g10025.1	*CqFTL5*		
	B	AUR62006619	*CqFTL5*	Chr05	77596526-77601590 (-strand)	528	176	*BvFT2*	EL10Ac4g10025.1	*CqFTL4*		
	A	AUR62026245	*CqFTL6*	Chr14	23254495-23258984 (+strand)	525	175	*BvFTL3*	EL10Ac6g13314.1	*CqFTL9*		
	B	AUR62026433	*CqFTL7*	Chr06	69532880-69534843 (+strand)	522	174					
	A	AUR62026237	*CqFTL8*	Chr14	22669640-22670374 (+strand)	315	105					
	B	AUR62026437	*CqFTL9*	Chr06	68781713-68782414 (+strand)	315	105			*CqFTL6*		
	B	AUR62026436	*CqFTL10*	Chr06	69126739-69127582 (+strand)	339	113					
	B	AUR62026435	*CqFTL11*	Chr06	69423321-69434601 (+strand)	792	264					
**TFL**	B	AUR62009771	*CqTFL1*	Chr18	26895130-26896703 (-strand)	504	168	*BvTFL1, BvTFL2*	EL10Ac7g15814.1, EL10Ac7g16930.1	*CqTFL3*	4:2	7:3
	A	AUR62039217	*CqTFL2*	Chr07	111421421-111422986 (+strand)	513	171					
	A	AUR62039216	*CqTFL3*	Chr07	111401972-111409400 (+strand)	792	264	*BvTFL1*	EL10Ac7g15814.1	*CqTFL1*		
	A	AUR62033497	*CqTFL4*	Chr07	8662902-8665663 (+strand)	519	173	*BvTFL2*	EL10Ac7g16930.1	*CqTFL5*		
	B	AUR62028545	*CqTFL5*	Chr17	43906953-43909946 (-strand)	519	173	*BvTFL2*	EL10Ac7g16930.1	*CqTFL4*		
	NA	AUR62021217	*CqTFL6*	Chr00	35800827-35802318 (+strand)	522	174	*BvBFT1*	EL10Ac3g05715.1	*CqTFL7*		
	A	AUR62016010	*CqTFL7*	Chr07	77891902-77893406 (-strand)	522	174	*BvBFT1*	EL10Ac3g05715.1	*CqTFL6*		

**Figure 1:**
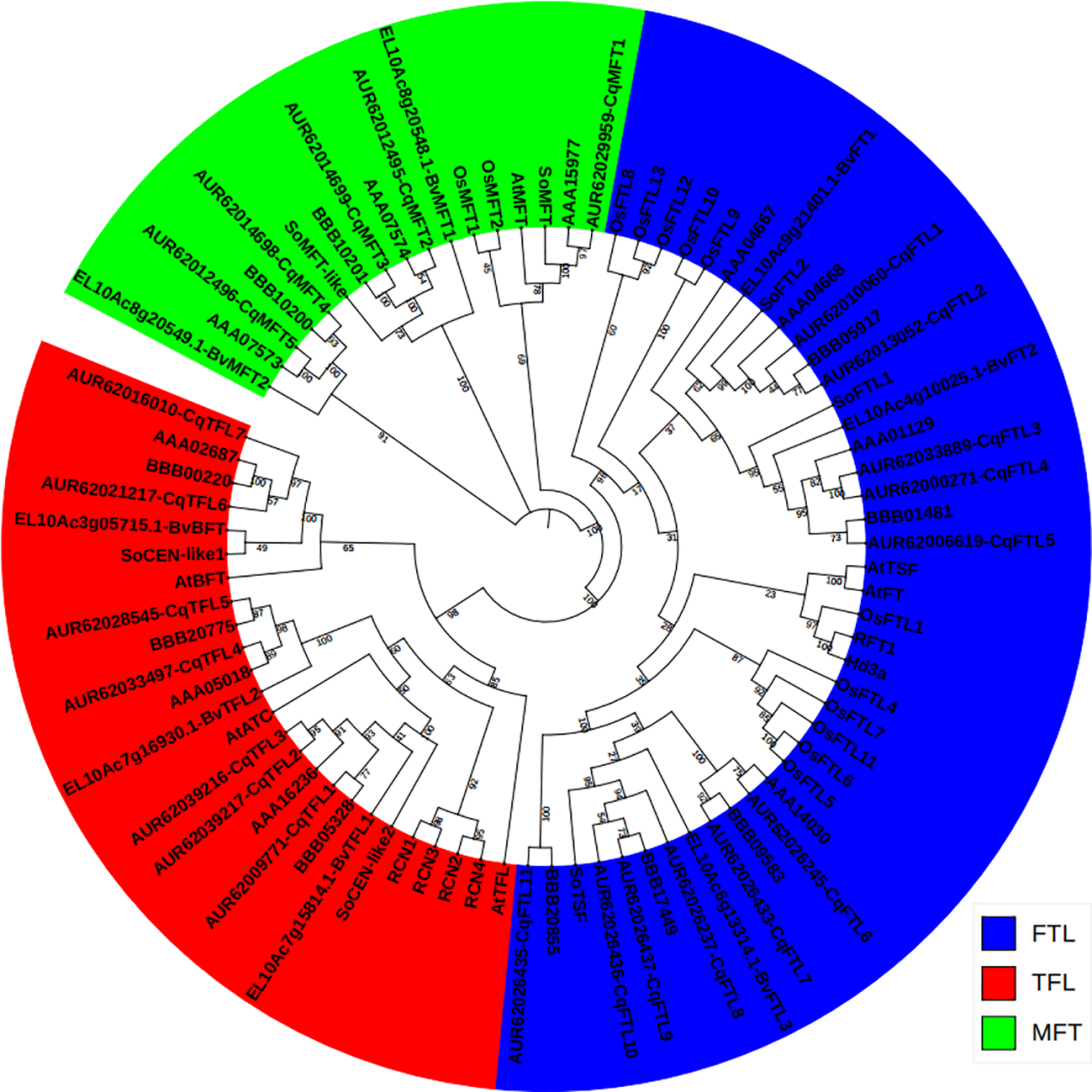
Phylogenetic relationship between the *PEBP* genes from *Chenopodium quinoa*, *C. pallidicaule*, *C. suecicum*, *Beta vulgaris*, *Spinacia oleracea*, *Arabidopsis thaliana* and *Oryza saltiva*. The PEBP protein sequences were downloaded from Phytozome V13 database. *MFT*, *FT* and *TFL* subclades are colored in green, blue and red, respectively. A total of 83 protein sequences were aligned using CLASTALW in MEGA 11.0. The phylogenetic tree was constructed by MEGA 11.0 using the Neighbor-Joining method with a bootstrap of 1000. The tree is unrooted, bootstrap values are indicated on branches.

To evaluate the phylogenetic relationships between *PEBP* genes of quinoa, and closet relatives of its diploid ancestors, *C. pallidicaule* (AA) and *C. suecicum* (BB), and its relatives in Amaranthaceae family, spinach (*Spinacia oleracea*) and sugar beet (*Beta vulgaris*), and model dicot and monocot plants, Arabidopsis (*Arabidopsis thaliana*) and rice (*Oryza sativa*), a Neighbor-Joining phylogenetic tree containing 83 sequences (**Supplementary File 1**) was inferred using MEGA 11.0 (Tamura et al., 2021). As displayed in **Figure 1**, 23 quinoa *PEBP* genes were sorted into three major subfamilies. Quinoa contains 5 *MFT* clade members, named *CqMFT1* to *CqMFT5*, 11 *FT-like* (*FTL*) clade members, named *CqFTL1* to *CqFTL11*, 7 *TFL1-like* (*TFL*) clade members, named *CqTFL1* to *CqTFL7* (**Figure 1**). *C. pallidicaule* contains 3 *MFT*s, 4 *FTL*s and 3 *TFL*s, and *C. suecicum* contains 2 *MFT*s, 5 *FTL*s and 3 *TFL*s (**Figure 1**).

**Figure 2:**
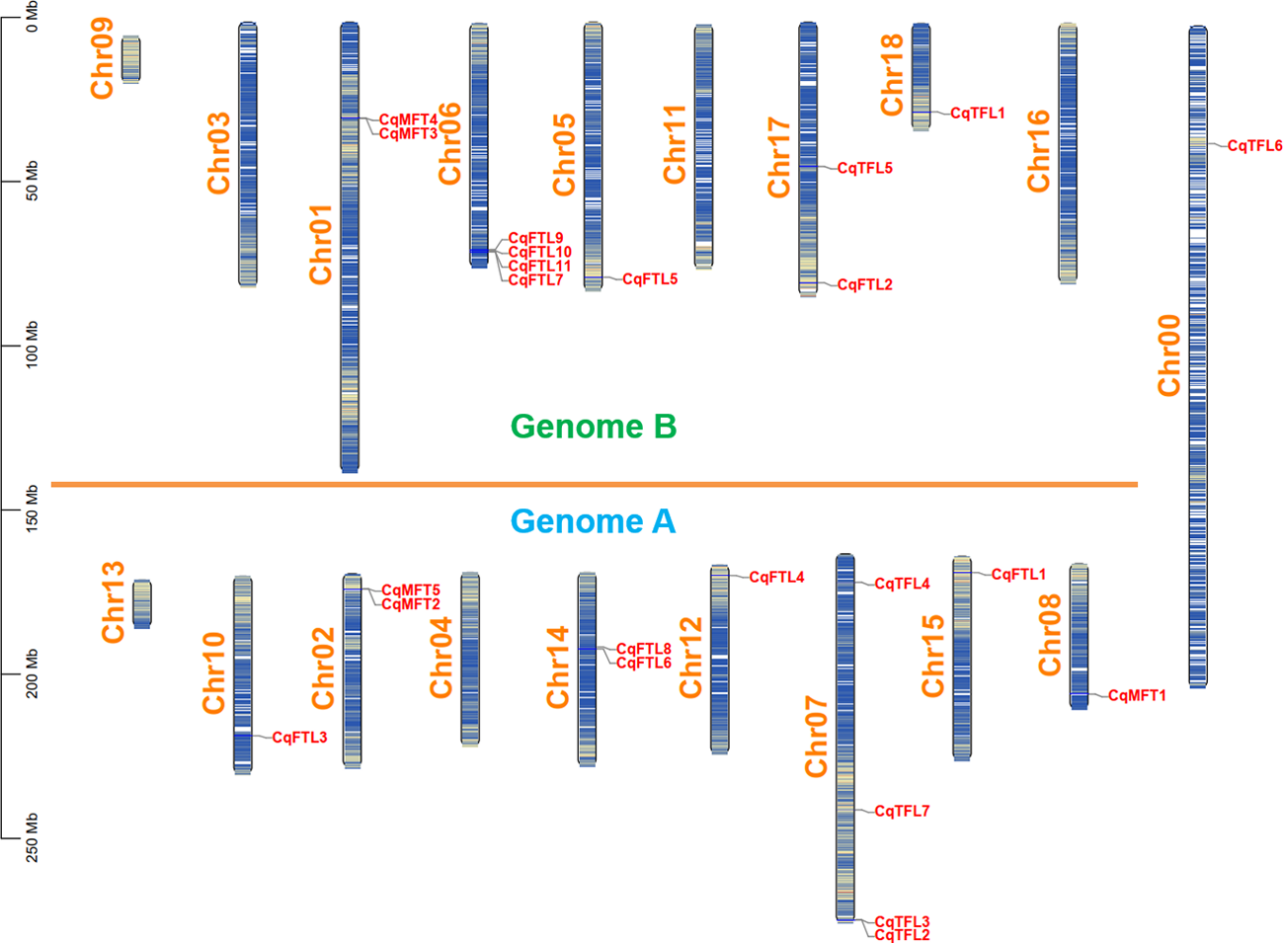
Physical location of *PEBP* genes on quinoa chromosomes. Chromosome segments colored in white and blue indicate high and low gene densities, respectively. All the quinoa chromosomes are divided into sub-genome A and B with the exception of Chr00.

Then, we drew a chromosomal location map of *PEBP*s. As illustrated in **Figure 2**, 23 *PEBP* genes are distributed on 12 chromosomes with the exception that *CqTFL6* is anchored on Chr00. Sub-genome A and sub-genome B have nearly equal number of *PEBP*s. Chr06 of sub-genome B and Chr07 of sub-genome A contain the largest number of *CqFTL*s and *CqTFL*s, respectively.

### Gene structure and conserved motifs of *PEBP* genes

**Figure 3:**
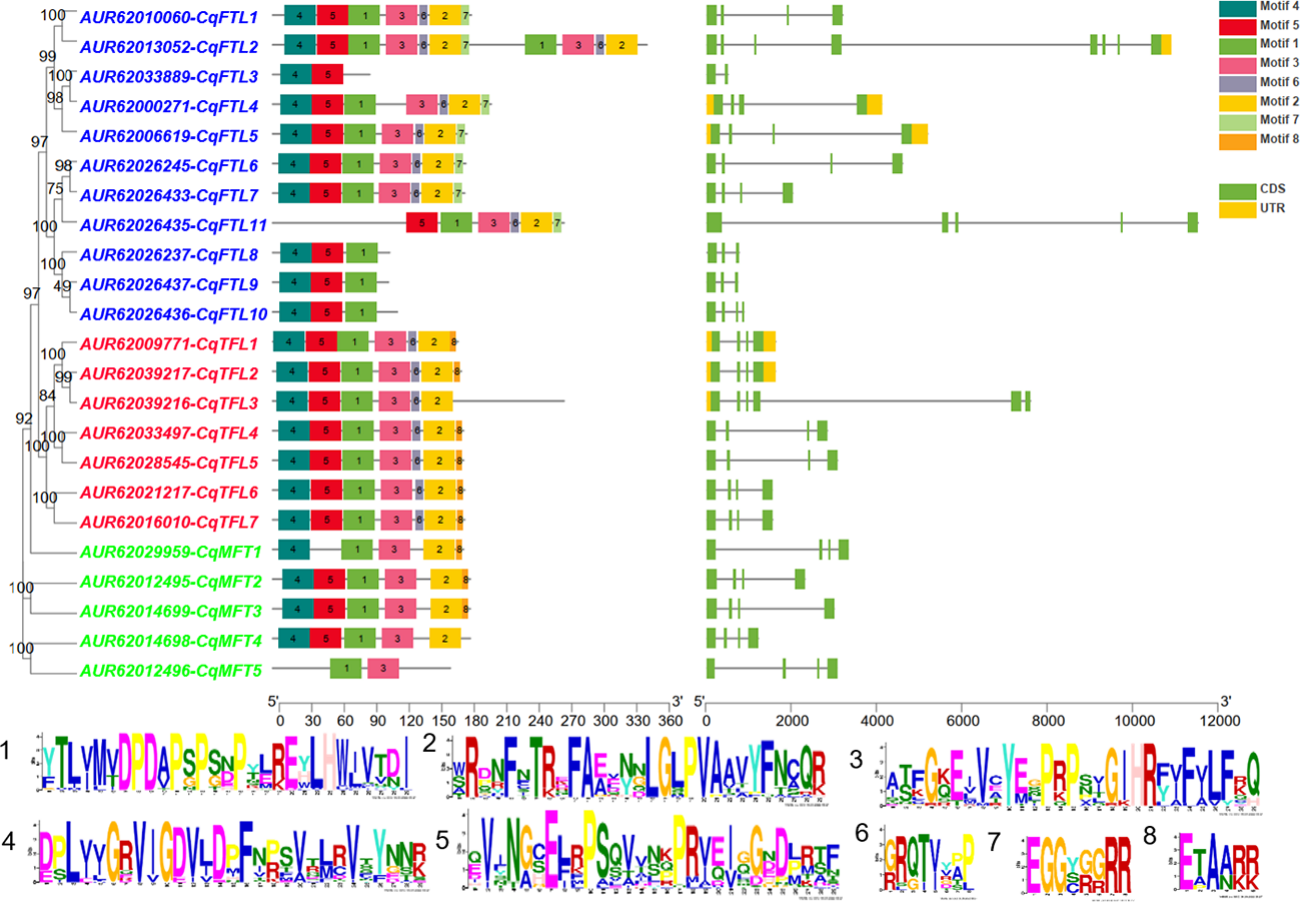
Diagram shows the conserved amino acid motifs and gene structure of *PEBP* genes in quinoa. The conserved motifs were identified by MEME. A total of 8 motifs were discovered among all *PEBP* genes. Different motifs are indicated by the colored boxes. Motif 1 to motif 5 are appeared in most PEBP proteins. Amino acid sequences of the conserved motifs are listed below. The identities of *FT*, *TFL* and *MFT* subclade genes are colored in blue, red and green in phylogenetic tree at the left side. Exons, introns and untranslated regions (UTRs) of *PEBP* genes are denoted by green, yellow boxes and dark lines, respectively.

Diagram of *PEBP* gene structures shows that, out of 23 *PEBP*s, most (17 of 23) harbor 4 exons and 3 introns (**Figure 3**). *CqFTL2* contains the most (8 exons and 7 introns), whereas *CqFTL3* contains the least (2 exons and 1 intron) number of exons and introns (**Figure 3**). MEME program was used to identify the conserved motifs in quinoa PEBP proteins. A total of 8 conserved motifs were identified in PEBP proteins (**Figure 3**). Motif composition schematic analysis indicates that motif 1, 2, 3, 4 and 5 were constitutively occurred in most of the *PEBP* genes (**Figure 3**). Yet, several PEBP proteins lack some specific motifs. For example, CqFTL2 lacks motif 1, 2 and 3; CqFTL8, CqFTL9 and CqFTL10 lack motifs 2 and 3; CqFTL11 lacks motif 4; CqMFT1 lacks motif 5; CqMFT4 lacks motifs 2, 4, and 5 (**Figure 3**). In addition, we found that motif 6 was occurred in most of the *FTL* and *TFL* subfamilies, whereas was absent in *MFT* subfamily (**Figure 3**). Motif 7 instead of motif 8 was possessed by most of the *FTL* subfamily members, while motif 8 was exclusively occurred in majority of *TFL* and *MFT* subfamilies (**Figure 3**). As PEBP proteins usually interact with other proteins (Taoka et al., 2013; Tsuji et al., 2013), such as FD and 14-3-3 proteins, to exert their roles, we speculated that these distinct motif compositions of quinoa PEBPs may result in varied protein-protein interactions and function diversities.

**Figure 4:**
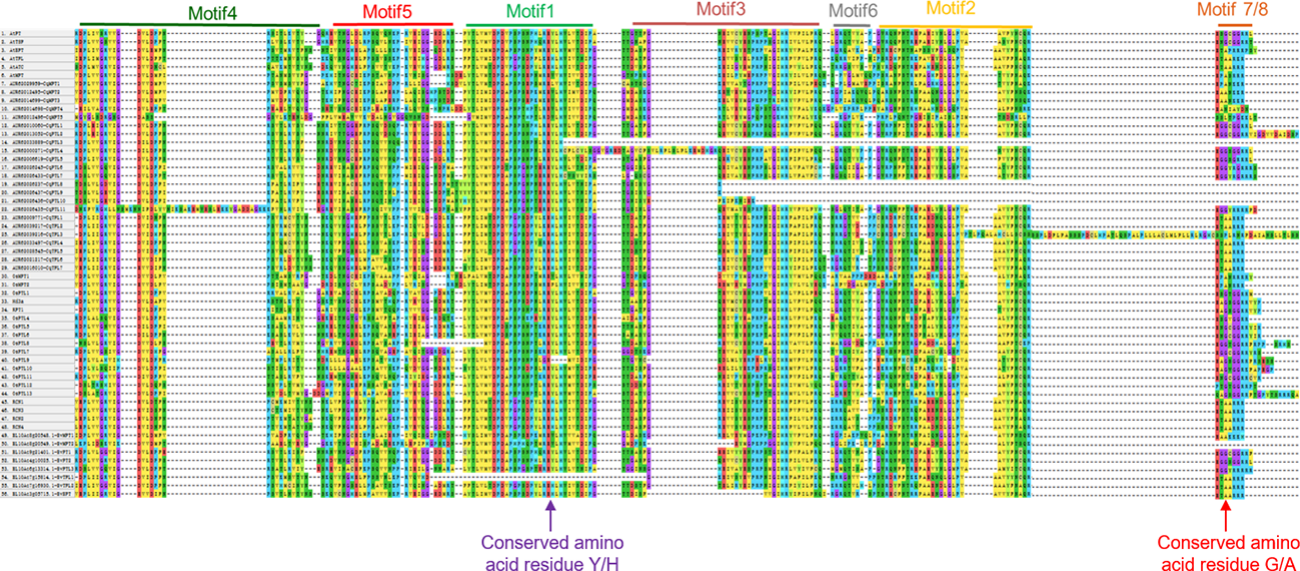
Multiple sequence alignment of the PEBP proteins from different plants. Sequences numbered with 1 to 6, 7 to 29, 30 to 48 and 49 to 56 stand for *PEBP* genes from Arabidopsis, rice, quinoa and sugar beet, respectively. The conserved motif 1 to motif 5 are indicated at the top of aligned sequences. Red arrow indicates the key conserved amino acid residue that determines *FT* and *TFL* subclade functions.

Multiple sequence alignment suggests that the conserved amino acid residue tyrosine (Y) or histidine (H) in motif 1 is a key site distinguishing *FTL* and *TFL* clades (**Figure 4**), in consistent with the findings in PEBP families of other plants (Wang et al., 2015). Besides, as the occurrences of motif 7 and motif 8 in *FTL* and *TFL*/*MFT* subfamilies are mutually exclusive (**Figure 4**), we speculated that in these two motifs there may be specific amino acid residues distinguishing *FTL* and *TFL*/*MFT* clades. As expected, we found that, the third amino acid residue in motif 7 and 8 was Glycine (G) in *FTL* genes, whereas it was Alanine (A) in *TFL* and *MFT* genes (**Figure 4**). Thus, this key site (G/A) may provide as a novel site for investigation of the converse functions of *FTL* and *TFL*/*MFT* clades.

### Gene duplication, collinearity and evolutionary history of *PEBP*s

**Figure 5:**
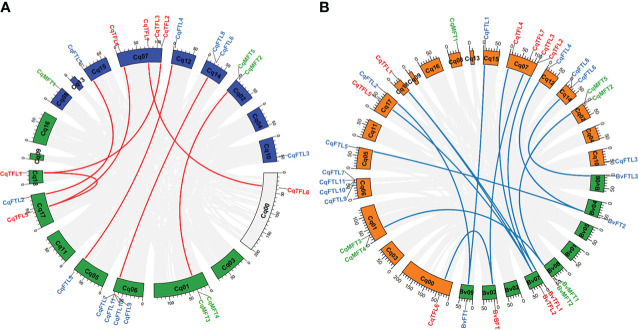
Circos plots shows the syntenic relationships between *PEBP*s from sub-genome A and B of quinoa and between *PEBP*s of quinoa and sugar beet. **(A)** Schematic representation of the syntenic relationships between homoeologous *PEBP*s of sub-genome A (blue) and B (green). Gray lines connect the homoeologous gene pairs of different chromosomes. Homoeologous *PEBP*s in quinoa are linked by red lines. **(B)** Syntenic relationships between the homoeologous *PEBP*s of quinoa and sugar beet. Chromosomes of quinoa and sugar beet are colored in green and orange, respectively. Gray lines connect the homoeologous gene pairs of quinoa and sugar beet. Blue lines connect the homoeologous *PEBP* gene pairs of quinoa and sugar beet.

Diploid sugar beet contains 8 *PEBP* genes in the genome. As a close relative of sugar beet, theoretically tetraploid quinoa (AABB) should has doubled number of *PEBP* homologs. However, we identified 23 *PEBP*s, nearly 3 times of that in *B. vulgaris* (**Table 1**). We wondered what caused the disproportional *PEBP* family expansion. Gene number ratios between quinoa and sugar beet in *MFT* and *TFL* clades are 5:2 and 7:3, respectively (**Table 1**). Surprisingly, gene number ratio in *FTL* clade is 11:3 (**Table 1**), remarkably higher than the plausible ratio 2:1. We analyzed the collinearities of *PEBP* genes between sub-genomes, and found that a total of 7 orthologous gene pairs from sub-genome A and B, including *CqMFT4*/*CqMFT5*, *CqFTL1*/*CqFTL2*, *CqFTL4*/*CqFTL5*, *CqFTL6*/*CqFTL9*, *CqTFL1*/*CqTFL3*, *CqTFL4*/*CqTFL5* and *CqTFL6*/*CqTFL7*, displayed collinear relationships (**Table 1**; **Figure 5A**). In addition, *CqTFL1* and *CqTFL5* also displayed collinearity (**Figure 5A**), indicating inner sub-genome duplication. Collinearity analysis between quinoa and sugar beet genomes showed that, 13 out of 23 *PEBP* genes of quinoa were orthologous to 7 out of 8 *PEBP*s of sugar beet (**Table 1**; **Figure 5B**). Due to the allotetraploidy of quinoa genome, most of the sugar beet *PEBP*s have two sister genes, from quinoa sub-genome A and sub-genome B, respectively. There are 7 orthologous clusters, including *CqMFT4*/*CqMFT5*-*BvMFT1*, *CqFTL1*/*CqFTL2*-*BvFT1*, *CqFTL4*/*CqFTL5*-*BvFT2*, *CqFTL6*-*BvFTL3*, *CqTFL1*/*CqTFL3*-*BvTFL1*, *CqTFL1*/*CqTFL4*/*CqTFL5*-*BvTFL2* and *CqTFL6*/*CqTFL7*-*BvBFT1* (**Table 1**; **Figure 5B**). Most of these orthologous groups are in line with the gene pairs from sub-genome A and sub-genome B. There are 3 copies of quinoa *TFL* gene in the *CqTFL1*/*CqTFL4*/*CqTFL5*-*BvTFL2* cluster (**Figure 5B**), probably rising from the segmental duplication events between Chr17 and Chr18 in sub-genome B (**Figure 5A**).

The rest of *PEBP*s, including *CqMFT1*, *CqMFT2*, *CqMFT3*, *CqFTL3*, *CqFTL7*, *CqFTL8*, *CqFTL10*, *CqFTL11* and *CqTFL2*, lack syntenic regions. We analyzed the gene physical locations, and found some of them were distributed in close vicinity to the other *PEBP* genes (**Table 1**; **Figure 2**). Among those genes, 4 tandem repeats were found, including *CqMFT3*/*CqMFT4* on Chr01, *CqMFT2*/*CqMFT5* on Chr02, *CqFTL9*/*CqFTL10*/*CqFTL11/CqFTL7* on Chr06 and *CqTFL2*/*CqTFL3* on Chr07 (**Table 1**; **Figure 2**). Notably, all these tandem repeats are located on distal telomeric ends of chromosomes (**Figure 5A**), indicating possible higher frequency of tandem duplications in distal telomeric segments. In general, these evidences indicated that tandem duplication is the major mechanism underlying *PEBP* family expansion.

Then, we analyzed the rise of gene duplication of *PEBP*s by combining phylogenetic analysis with collinearity analysis. According to the genome map of sugar beet, *BvMFT1* and *BvMFT2* are in tandem repeat location. Meanwhile, we found the orthologs, in the relatives of sub-genome A (AAA07574/AAA07573 in *C. pallidicaule*) and sub-genome B (BBB10201/BBB10201 in *C. suecicum*), are also in tandem repeat locations. Thus, we deduced that the tandem duplications of *CqMFT3/CqMFT4* and *CqMFT2/CqMFT5* happened before the divergence of the ancestor of *Chenopodium* from sugar beet, far before the tetraploidization of quinoa. Though *CqMFT1* was in non-synteny with sugar beet and *C. suecicum*, however, it is sister to the *MFT* genes of Arabidopsis, and *C. pallidicaule* and spinach. Phylogenetic analysis indicated that the tandem duplication of *CqTFL2*/*CqTFL3* was happened after the ancestor of sub-genome A diverged from *C. pallidicaule*. *CqFTL3*, a sister of *CqFTL4* in the phylogenetic tree, but is in non-syntenic region and lacks 4 motifs of *CqFTL4*, indicating that gene amplification may occur through transposable elements. *CqFTL8*, despite located nearby *CqFTL6*, but was not detected as a tandem repeat with *CqFTL6* (**Figure 5A**). Meanwhile, *CqFTL8* is in non-syntenic region and lacks motif 3, 6, 2, 7 compared with the other *CqFTL* genes (**Figure 3**), indicating it might arise from small-scale transposition. Synteny was found in *CqFTL9* of sub-genome A and *CqFTL6* of sub-genome B (**Figure 5A**), whereas only *CqFTL6*, rather than *CqFTL9*, has synteny with *BvFTL3* of sugar beet (**Figure 5B**), suggesting the tandem duplication in *CqFTL9*/*CqFTL10*/*CqFTL11/CqFTL7* possibly was generated after the divergence of the ancestor of sub-genome B from sugar beet.

### Distribution of *cis-*acting elements in *PEBP* promoters

**Figure 6:**
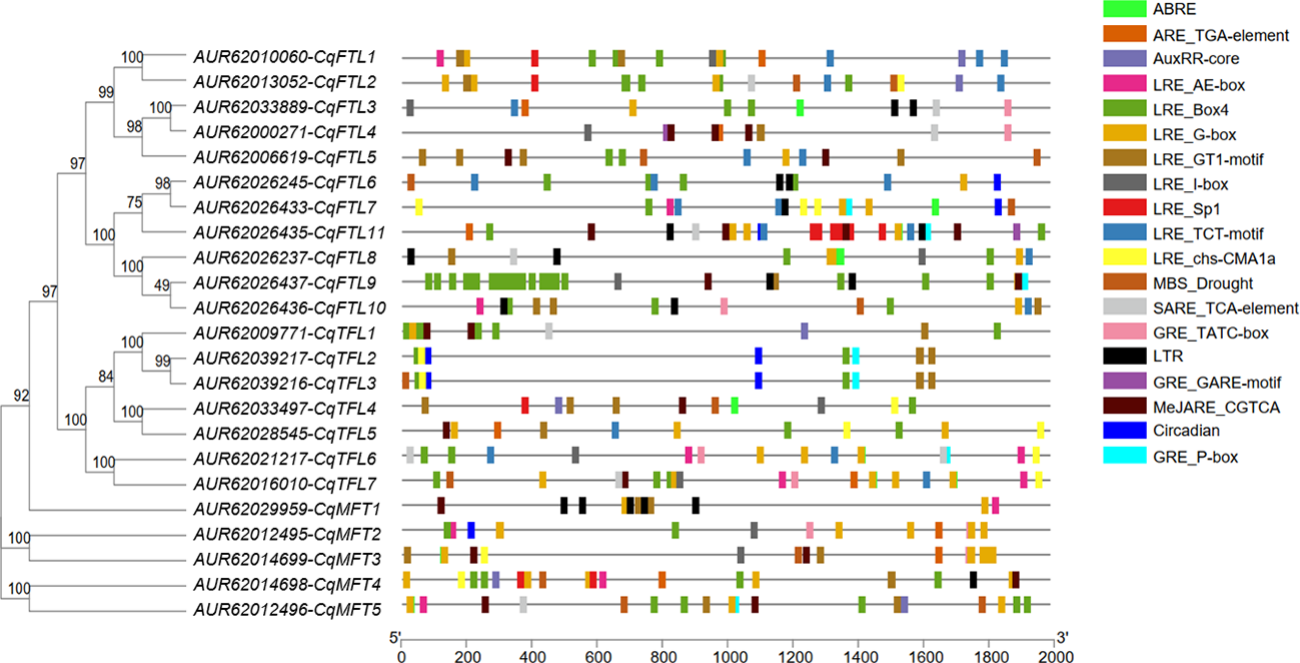
Illustration shows the physical locations of various *cis-*acting elements in the promoter regions of *PEBP*s. Promoter sequences of quinoa *PEBP*s were submitted to PlantCARE database to identify various *cis*-acting elements. Boxes with different colors indicate various *cis-*acting elements located in the promoter regions of *PEBP*s. Abbreviations and sequences: ABRE, abscisic acid responsive element, ABRE: ACGTG; ARE, auxin-responsive element, ARE_TGA-element: AACGAC; AuxRR, auxin responsive element, AuxRR-core: GGTCCAT; LRE, light responsive element, LRE_AE-box: AGAAACAA, LRE_Box 4: ATTAAT, LRE_G-box: CACGTG, LRE_GT1-motif: GGTTAA, LRE_I-box: AGATAAGG, LRE_Sp1: GGGCGG, LRE_TCT-motif: TCTTAC, LRE_chs-CMA1a: TTACTTAA; MBS, MYB binding site, MBS_Drought: CAACTG; SARE, salicylic acid responsive element, SARE_TCA-element: CCATCTTTTT; GRE, gibberellin-responsive element, GRE_TATC-box: TATCCCA, GRE_GARE-motif: TCTGTTG, GRE_P-box: CCTTTTG; LTR, low-temperature responsive element, CCGAAA; MeJARE, MeJA-responsive element, MeJARE_CGTCA: CGTCA; Circadian, circadian regulatory element, Circadian: CAAAGATATC.

*Cis-*acting elements in gene promotor have important regulatory roles mediating transcriptional activation and repression, and various *cis-*acting elements controlling specific progresses have been identified. To predict and understand the versatile functions of *PEBP* genes, we explored the *cis-*acting elements in promotors. Two kilo base pairs upstream of the translational start site of *PEBP* genes were submitted to PlantCARE database for *cis-*acting elements prediction. As displayed in **Figure 6**, various *cis-*acting elements, related to light, phytohormones, cold, drought and circadian clock responses, were identified. Light responsive elements (LREs) take a great proportion among various *cis-*acting elements in *PEBP* promoters, suggesting that *PEBP*s may be tightly associated with light biological events. A total of 8 kinds of LREs were identified. LRE_Box4 and LRE_G-box were the two major elements distributed in all *PEBP* promoters. Some *cis-*acting elements showed gene-specific distribution patterns. *CqFTL8* promoter harbors the most abundant LRE_Box4. *CqFTL11* promoter harbors more LRE_Sp1. LRE_G-box rather than LRE_Box4 was more abundant in the promoters of *MFT* clade. More low-temperature responsive elements (LTRs) were enriched in *CqMFT1* promoter, indicating the possible roles of *CqMFT1* in sensing cold stress. Circadian regulatory elements (Circadian) were only distributed in the promoters of *CqFTL7*, *CqFTL11*, *CqMFT2*, *CqTFL2* and *CqTFL3*. Abscisic acid responsive elements (ABREs) were abundantly located in promoters of *CqMFT2* and *CqMFT3*, indicating these two gene may be involved in ABA signaling. In addition, we noticed distributions of the *cis-*acting elements in promoters of some gene pairs were also in synteny. The distributions of ABRE, AuxRR-core, LRE_Box4, LRE_G-box, LRE_GT1-motif, LRE_Sp1 and LRE_TCT-motif in *CqFTL1* promoter were in synteny with that in *CqFTL2* promoter. This is in line with the collinearity between these two genes. The distributions of the *cis-*acting elements including Circadian, GRE_P-box, LRE_Box4, LRE_chs-CMA1a and LRE_GT1-motif in *CqTFL2* promoter were in highly collinear relationship with that in *CqTFL3* promoter. This may be caused by the tandem duplication of *CqTFL2*/*CqTFL3*. The specific distributions of *cis-*acting elements in *PEBP* promoters suggest their possible roles in different biological events.

### Tissue-specific expression analysis of *PEBP*s

**Figure 7:**
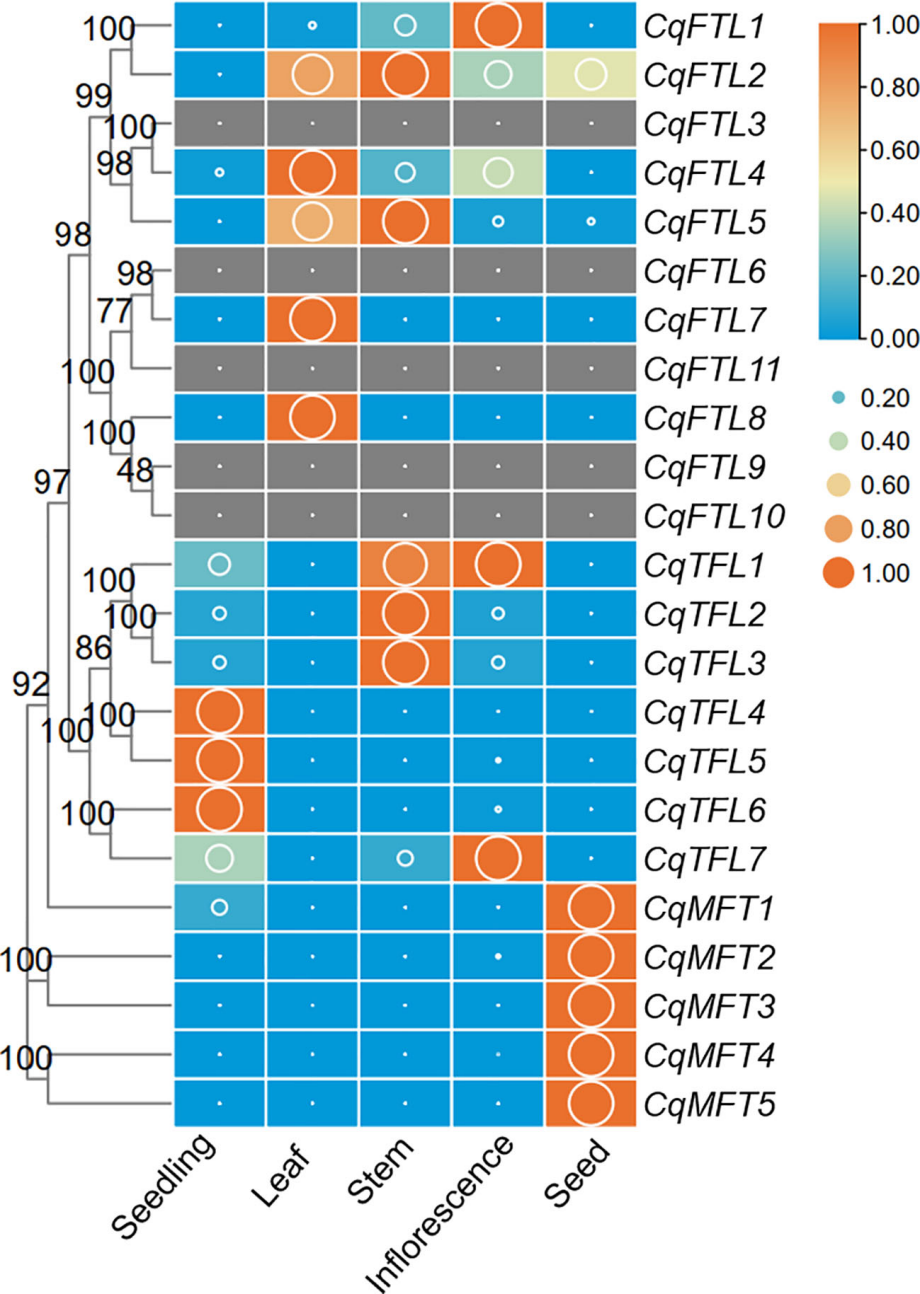
Expression profiles of *PEBP* genes in 5 quinoa tissues. The expression value is denoted by FPKM values (cutoff=0.1) generated by using the RNA-seq data from Zou et al. (Zou et al., 2017). Each expression value was generated from three replicates. Orange boxes with larger-size bubbles indicate higher expression levels. Expressions in various tissues of the same gene were normalized to 0 to 1 by row.

To characterize the expression patterns of *PEBP*s in various quinoa organs, we analyzed the RNA-seq data and compared the FPKM values of 23 *PEBP* genes in seedling, leaf, stem, inflorescence and seed. Out of 23 *PEBP* genes, 18 were expressed in at least one organ tissue (**Figure 7**; **Supplementary File 2**). Generally, expressions of *PEBP*s in various tissues were clade-specific. *FTL* clade genes were more enriched in leaf, stem and inflorescence, and *TFL* clade genes were abundant in seedling, stem and inflorescence, whereas expressions of the *MFT* clade genes were relatively higher in seed (**Figure 7**). It seems like that expressions of the *MFT* clade genes are more conserved, and all the *MFT* gene pairs were expressed in similar patterns (**Figure 7**). By contrast, expressions of the orthologous gene pairs in *FTL* and *TFL* clades were likely differing (**Figure 7**). For *CqFTL1*/*CqFTL2*, *CqFTL1* was highly enriched in inflorescence, whereas *CqFTL2* was ubiquitously expressed in all tissues (**Figure 7**). For *CqFTL4*/*CqFTL5*, *CqFTL4* was expressed mainly in leaf, and *CqFTL5* was abundant in leaf and stem (**Figure 7**). Another branch containing *CqFTL7* and *CqFTL8* was expressed specifically in leaf (**Figure 7**). For *CqTFL1*/*CqTFL3*, higher expressions of *CqTFL1* were detected in both stem and inflorescence, and *CqTFL3* was specifically abundant in stem (**Figure 7**). The duplicated gene of *CqTFL2*, *CqTFL3*, was also expressed mainly in stem (**Figure 7**). For *CqTFL6*/*CqTFL7*, *CqTFL6* was specifically expressed in seedling, whereas *CqTFL7* transcripts were abundant in seedling and inflorescence (**Figure 7**). *CqTFL4*/*CqTFL5* harbored similar expression patterns, both were specifically expressed in seedling. The differing expression patterns in gene pairs indicate the possible diversified roles of orthologs from sub-genome A and sub-genome B.

### The *PEBP*s involved in inflorescence branching

**Figure 8:**
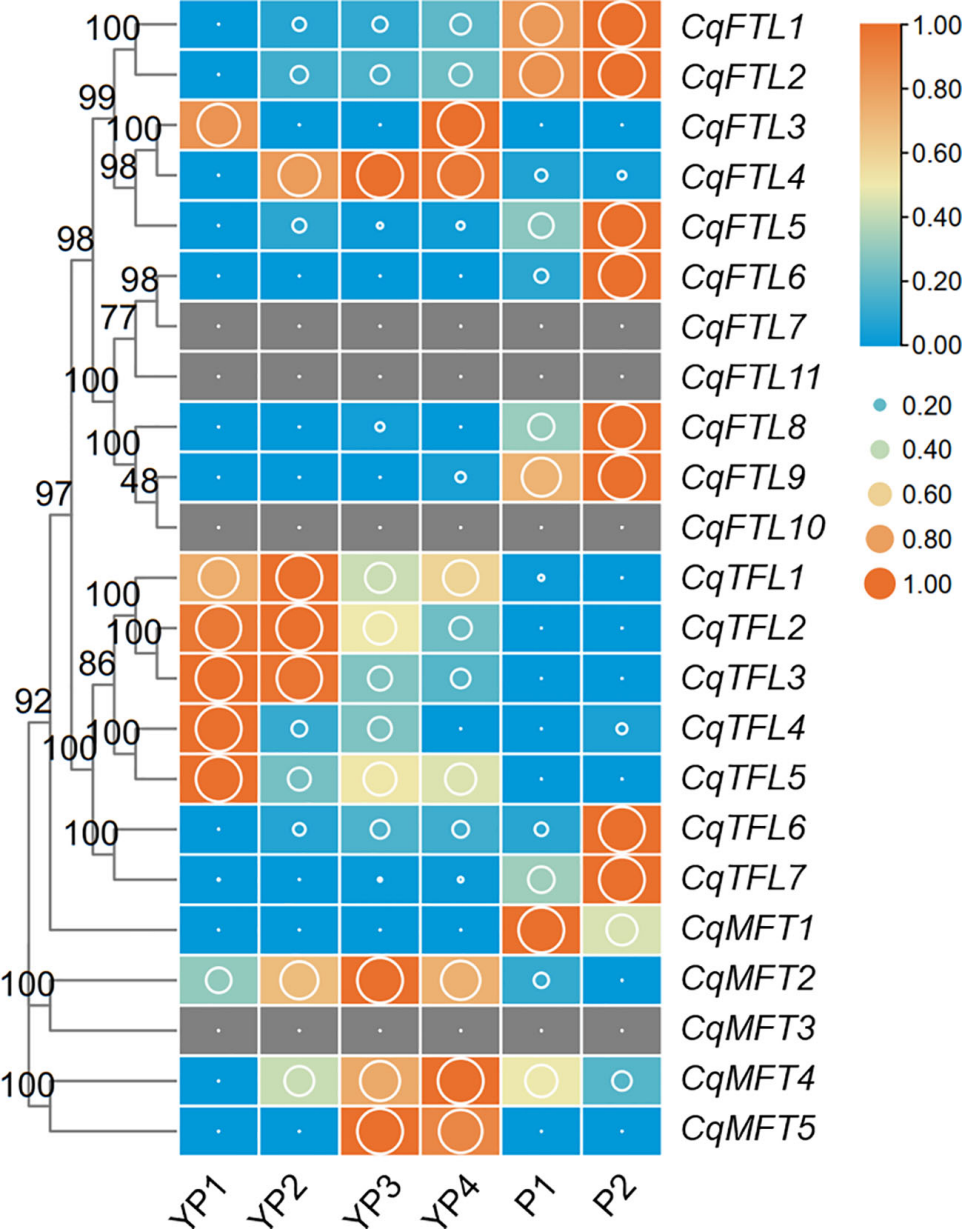
Expression of *PEBP* genes during quinoa inflorescence development. RNA-seq data of inflorescences sampled from 6 developmental stages was used to investigate the expression changes of *PEBP*s (Wu et al., 2019). Expression levels are denoted by FPKM values (cutoff=0.1). YP1 to YP4 indicates non-branching young panicles at 4 earlier developmental stages. P1 and P2 indicate branching panicles at 2 later developmental stages. Each expression value was generated from three replicates. Orange boxes with larger-size bubbles indicate higher expression levels. Expression levels at 6 stages of the same gene were normalized to 0 to 1 by row.

Branching of inflorescence is a key factor influencing plant architecture and yield. Numerous evidences have suggested the important roles of *PEBP* genes in inflorescence branching and yield control (Teo et al., 2014; Benlloch et al., 2015). To investigate which quinoa *PEBP* members are potential regulator for panicle branching, we analyzed the expression changes of *PEBP*s across 6 developmental stages, before and after panicle branching. Expressions of 19 *PEBP*s were detected in at least one stage (**Figure 8**; **Supplementary File 2**). Differing from the diversified expressions in tissues above, most of the orthologous gene pairs in *FTL* and *TFL* clades showed similar expression patterns. For *FTL* clade, with the exception that *CqFTL3* and *CqFTL4* were expressed ubiquitously at non-branching stages (YP1 to YP4) (**Figure 8**), expressions of *CqFTL1*/*CqFTL2*, *CqFTL5*, *CqFTL6*/*CqFTL9* and *CqFTL8*, ascended with the development of inflorescence and were abundant at branching stages (P1 and P2) (**Figure 8**). For *TFL* clade, expression levels of gene pairs *CqTFL1*/*CqTFL3*, *CqTFL4*/*CqTFL5*, and the duplicated gene of *CqTFL3*, *CqTFL2* were relatively higher at non-branching stages (YP1 and YP2) while descended with the development of inflorescence (**Figure 8**), whereas the gene pairs *CqTFL6*/*CqTFL7* were relatively abundant in branching stages (P2) (**Figure 8**). Most of the *MFT* clade genes were ubiquitously expressed across all stages (**Figure 8**). The specific expressions of *CqTFL1*/*CqTFL2*/*CqTFL3* and *CqTFL4*/*CqTFL5* at non-branching stages, and the enrichment of *CqFTL1*/*CqFTL2*, *CqFTL5*, *CqFTL6*/*CqFTL9*, *CqFTL8* and *CqTFL6*/*CqTFL7* at branching stages, suggest that those *PEBP*s may participate in panicle architecture regulation.

### The principal *PEBP*s involved in seed germination

**Figure 9:**
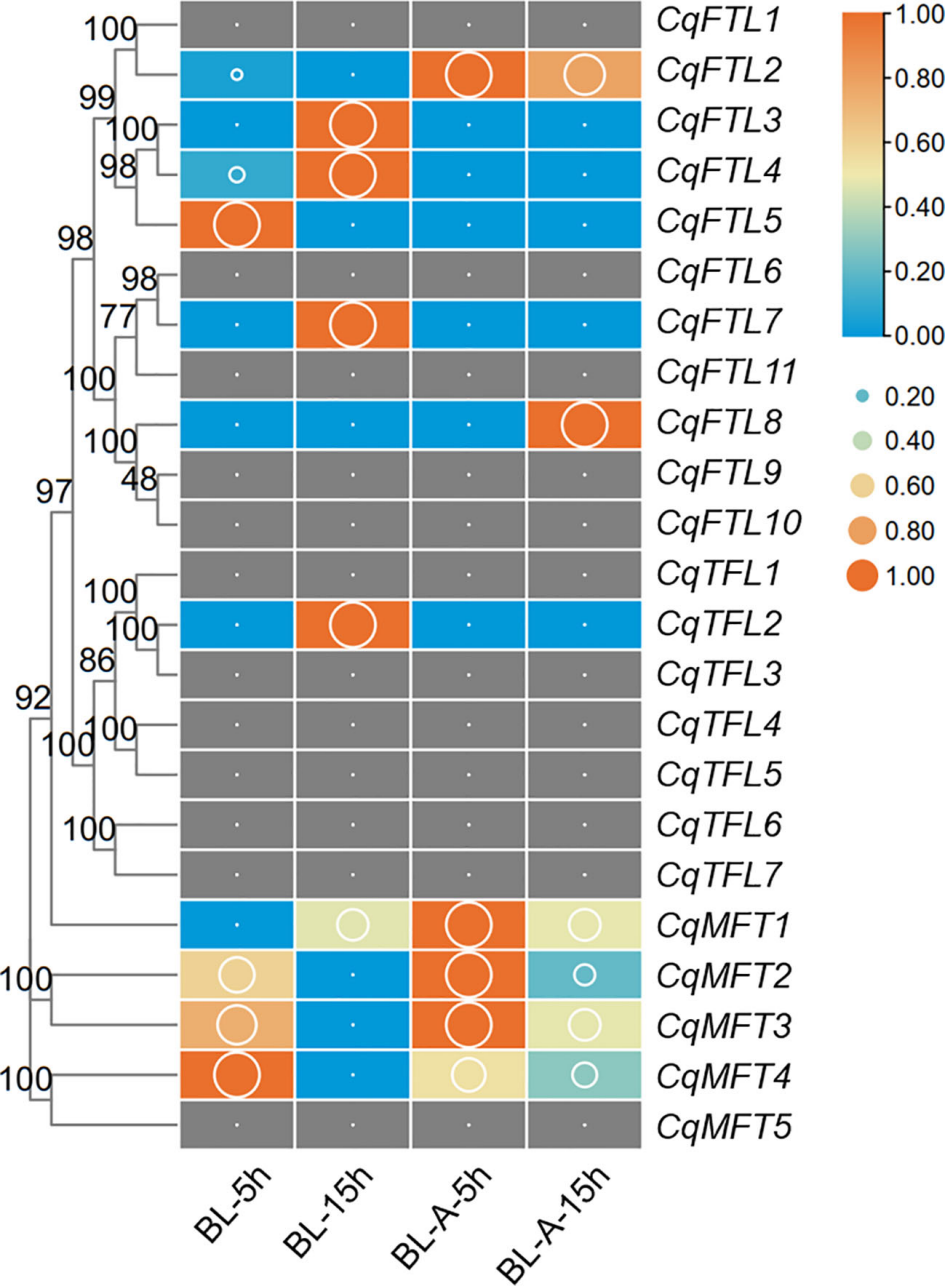
Expression changes of *PEBP*s during seed germination. RNA-seq data of control and ABA-treated seeds at 5h and 15h after imbibition was used to investigate the expression levels denoted by FPKM values (cutoff=0.1) (Wu et al., 2020). BL-A-5h/15h and BL-5h/15h indicate the seeds of cultivar “BL” with or without ABA treatment. Each expression value was generated from three replicates. Orange boxes with larger-size bubbles indicate higher expression levels. Expression values in various seed samples of the same gene were normalized to 0 to 1 by row.

Pre-harvest sprouting (PHS) is a knotty problem that influences the yield and nutritional qualities of quinoa (Wu et al., 2020). Understanding the mechanisms underlying seed germination will facilitate breeding PHS-resistant elites of quinoa. We investigated the expressions of *PEBP*s during seed germination before and after ABA treatment. A higher proportion of *MFT* clade genes (4 out of 5, 80%), whereas relative lower percentages of *FTL* clade (6 out of 11, 55%) and *TFL* clade genes (1 out of 7, 15%) were detected during seed germination (**Figure 9**; **Supplementary File 2**). We found *CqMFT2*, *CqMFT3* and *CqMFT4* were largely repressed as germination went on (**Figure 9**). Further, we found that the transcriptional changes of *CqMFT2*, *CqMFT3* and *CqMFT4* during germination were attenuated when treated by ABA (**Figure 9**). This is in agreement with the enrichment of the *cis-*acting element ABRE in *MFT* gene promoters, indicating those three genes may respond to ABA to regulate seed germination.

### Responses of *PEBP* genes to night break

**Figure 10:**
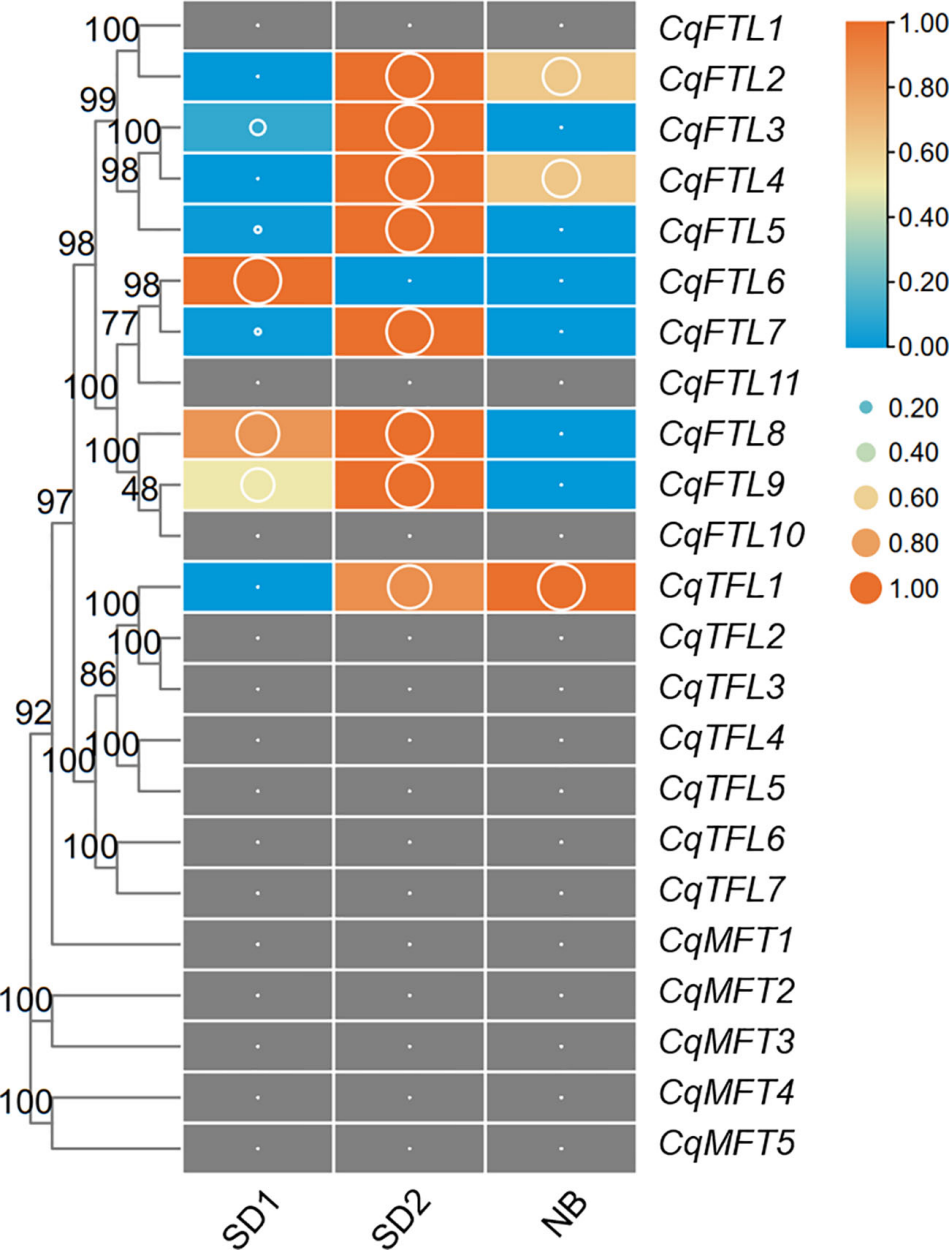
Investigation on the transcriptional changes of *PEBP*s in response to night break. Our previously published transcriptome data was used to. SD1 and SD2 indicate leaf samples collected from 14-day- and 26-day-old plants under short-day conditions. NB indicates the leaf sample of SD2 plants with 2d night-break treatment. Each expression value was generated from three replicates. Orange boxes with larger-size bubbles indicate higher expression levels. Expression values (cutoff=0.1) in various leaf samples of the same gene were normalized to 0 to 1 by row.

Night break (NB) has a repressive effect on the flowering of short-day plant (SDP) mostly by repressing florigen-encoding genes (Ishikawa et al., 2005). Like that in other SDPs, in our previous study we also noticed the repressive effects of NB on quinoa flowering. To know which *PEBP* genes may be involved in NB responses, we analyzed the transcriptome data of quinoa leaf samples collected from SD and NB conditions. As illustrated in **Figure 10**, *FT* clade genes were more active than *TFL* and *MFT* clade genes. A total of 9 *PEBP* genes were detected, of which 8 were *FTL* clade genes (**Figure 10**; **Supplementary File 2**). Obviously, *CqFTL3*, *CqFTL5*, *CqFTL7*, *CqFTL8* and *CqFTL9* were expressed at higher levels under SD, whereas were rapidly down-regulated by NB treatment (**Figure 10**). The opposed expression patterns of the 5 *FTL* genes under SD and NB indicate their possible positive effects in quinoa flowering. In addition, we noticed the differing responses to NB between orthologous gene pairs. For example, in *CqFTL4*/*CqFTL5*, *CqFTL5* was sensitive to NB, whereas *CqFTL4* was only slightly repressed by NB (less than 2 folds). In *CqFTL6*/*CqFTL9*, *CqFTL9* was largely repressed by NB, while *CqFTL6* were down-regulated regardless of light conditions.

### Diurnal expression analysis of *PEBP*s

**Figure 11:**
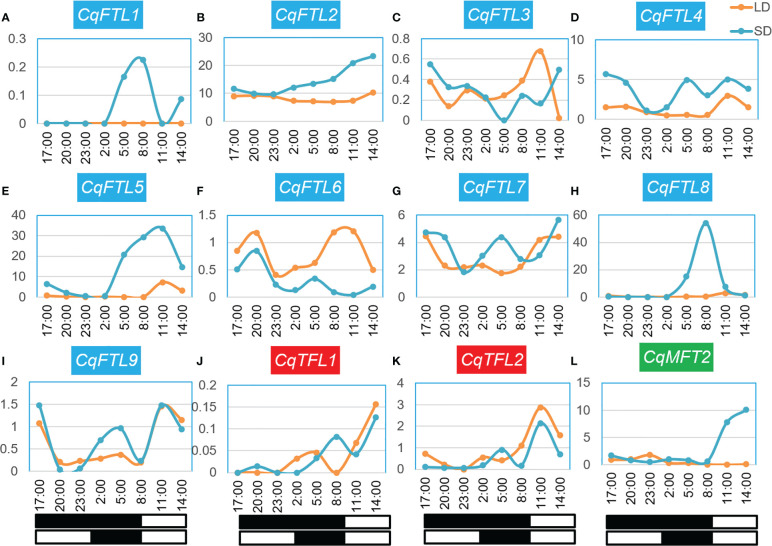
Diurnal expression patterns of *PEBP*s under short-day and long-day conditions. **(A-L)** Expression patterns of *CqFTL1*, *CqFTL2*, *CqFTL3*, *CqFTL4*, *CqFTL5*, *CqFTL6*, *CqFTL7*, *CqFTL8*, *CqFTL9*, *CqTFL1*, *CqTFL2* and *CqMFT2* under short-day and long-day conditions. Two-week-old plants grown under short-day and long-day conditions were used to collect the leaf samples, respectively, at 17:00, 20:00, 23:00, 02:00, 05:00, 08:00, 11:00 and 14:00. RNA-seq analysis was performed to investigate the expression patterns of *PEBP*s. White and black boxes indicate day and night time, respectively. Each expression value was generated from two replicates. Orange and blue curves stand for the expression levels of *PEBP*s under long-day and short-day conditions.

As quinoa belongs to SDPs, SD induces whereas long day (LD) represses quinoa flowering. To evaluate the relationships between *PEBP*s and day lengths, we performed a comprehensive analysis on *PEBP* expressions over 24 h under SD and LD. The quinoa plants were grown in growth chamber supplied with SD or LD. Before bolting stage, the top-fully-expanded leaves of two-week-old seedlings were collected for RNA-seq. We detected the expressions of 9 *FTL* clade genes, but only 2 *TFL* and 1 *MFT* clade genes were detected in leaves at this stage (**Figures 11A-L**; **Supplementary File 2**). The 24 h expression profile showed that, the expressions of several quinoa *PEBP* genes were diurnally rhythmic and differed under different day lengths (**Figures 11A, E, F, H, L**). Clearly, expression patterns between the orthologous gene pairs *CqFTL1*/*CqFTL2* (**Figures 11A, B**), *CqFTL4*/*CqFTL5* (**Figures 11D, E**), *CqFTL6*/*CqFTL9* (**Figures 11F, I**) were different. Expressions of *CqFTL1*, *CqFTL4*, *CqFTL5* and *CqFTL8* were considerably higher under SD than that under LD (**Figures 11A, D, E, H**). By contrast, *CqFTL6* was induced by LD rather than SD (**Figure 11F**). Expression levels of *CqFTL7* and *CqFTL9* were slightly higher under SD than that under LD (**Figures 11G, I**). Expression levels of *CqFTL3* were comparable between under SD and under LD (**Figure 11C**). *CqFTL1* and *CqFTL8* expressions were peaked at dawn time, and *CqFTL5* and *CqFTL9* were abundantly expressed at 2 h after dawn break (**Figures 11A, E, H, I**). *CqFTL2* was highly expressed throughout the day under both SD and LD (**Figure 11B**). The expressions of *CqTFL1* and *CqTFL2* were comparable between under SD and LD (**Figures 11J, K**). *CqMFT2* was induced by SD and peaked at the end of day (**Figures 11L**). Together, these results indicated that *CqFTL1*, *CqFTL4*, *CqFTL5* and *CqFTL8* may act as SD-type flowering inducer, whereas *CqFTL6* may act as LD-type flowering inducer. Recently, Patiranage et al. also investigated the diurnal expression patterns of *FTL* genes, but only limited to 6 *FTL* genes of quinoa at bolting stage (Patiranage et al., 2021). As florigen encoding *FT-like* genes are highly induced in leaves before floral transition, we chose to investigate the diurnal expressions at vegetative stage before bolting. The classical florigen genes are likely to diurnally expressed and are sensitive to night break (Ishikawa et al., 2005; Meng et al., 2011). By combining the responses to night break and photoperiodic expressions of quinoa *FTL* genes, we speculated that *CqFTL5* and *CqFTL8* are the major florigen-encoding genes in quinoa.

### Prediction of the putative TFs upstream of *CqFTL5* and *CqFTL8* by large-scale co-expression network analysis

**Figure 12:**
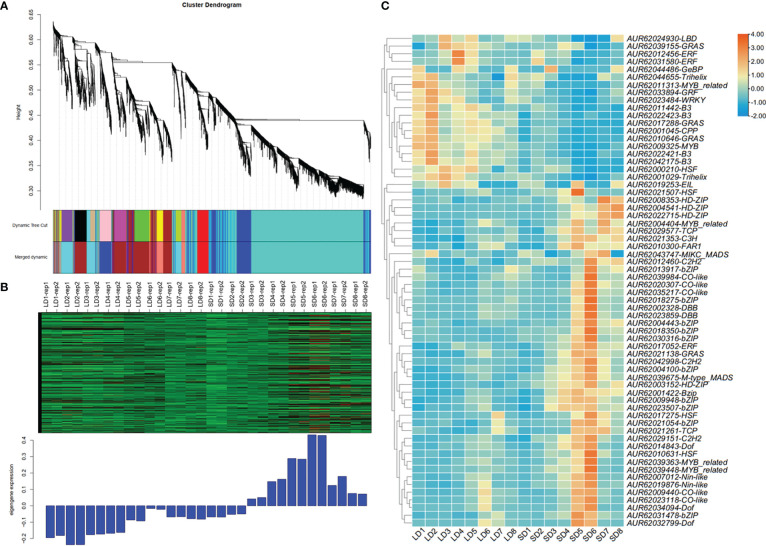
Weighted gene co-expression network analysis and transcription factor prediction methods were used to identify the co-expressed TFs with *CqFTL5* and *CqFTL8*. **(A)** All the expressed genes were sorted into 6 co-expressed modules by applying weighted gene co-expression network analysis. **(B)** *CqFTL5* and *CqFTL8* were clustered into blue module containing 934 co-expressed genes. SD1 to SD8, or LD1 to LD8, refers to the samples diurnally collected at 17:00, 20:00, 23:00, 02:00, 05:00, 08:00, 11:00 and 14:00. **(C)** Heatmap shows the expression levels of 64 TFs identified from blue module.

As floral integrators, *FT* genes usually are regulated by a complex network consisting of a lot of transcription factors (TFs) (Tsuji et al., 2013). To know which TFs may lay in the signal cascades upstream of *FT* genes in quinoa, WGCNA was performed to identify the co-expressed genes. The expression profiles of 32 samples covering 16 time points of SD and LD were used for WGCNA. A total of 6 co-expression modules were obtained. We found only *CqFTL5* and *CqFTL8* were predicted to occur in those modules. *CqFTL5* and *CqFTL8* were clustered into the same module-blue module, which contains 934 co-expressed genes. Then those genes were submitted to PlantTFDB v5.0 for TF prediction. A total of 64 TFs, 6.85% of blue module genes, were predicted to be co-expressed with *CqFTL5* and *CqFTL8*. A heatmap was drawn according to their expression profiles (**Figure 12**). Clearly, those TFs were clustered into two major clusters. One cluster displayed LD-repressive and SD-inducible expression patterns, consistent with that of *CqFTL5* and *CqFTL8*, whereas, the other cluster displayed opposed expression patterns (**Figure 12**). Thus, those TFs of the two clusters were predicted to be putative promoters and repressors of *CqFTL5* and *CqFTL8*. Notably, compared with the overall TF percentage of blue module genes, remarkable higher rates of family members were found in CO-like (CONSTANS), DBB (Double B-box), GeBP, HSF (Heat Stress Transcription Factors) and bZIP families (**Table 2**). *DBB* encodes for a double B-box zinc finger protein, and is regulated by circadian rhythm and participates in light signal transduction during photomorphogenesis. Previous studies indicated that *CO-like* encodes for a B-box zinc finger protein, and functions as an important mediator of circadian clock to control *FT* during floral transition (Putterill et al., 1995; Izawa et al., 2002). Thus, like that in other plants, the *CO-FT* signal pathway is presumably conserved in quinoa flowering. The central roles of MADS-box genes during floral transition have been extensively investigated (Kim et al., 2008; Ryu et al., 2009). We identified that two MADS-box genes were co-expressed with *CqFTL5* and *CqFTL8*. Together, we speculated that these TFs may modulate the transcription levels of *CqFTL5* and *CqFTL8* in leaves to control quinoa flowering time.

**Table 2:** The transcription factors co-expressed with *CqFTL5* and *CqFTL8* in blue module.

TF family	TF number in blue module	Number of TF family members	Percentage
B3	4	107	3.74%
bZIP	11	89	12.36%
C2H2	3	98	3.06%
C3H	1	79	1.27%
CO-like	5	14	35.71%
CPP	1	10	10.00%
DBB	2	8	25.00%
Dof	3	38	7.89%
EIL	1	9	11.11%
ERF	3	123	2.44%
FAR1	1	136	0.74%
GeBP	1	5	20.00%
GRAS	4	54	7.41%
GRF	1	11	9.09%
HD-ZIP	4	46	8.70%
HSF	4	30	13.33%
LBD	1	54	1.85%
MIKC_MADS	1	50	2.00%
M-type_MADS	1	53	1.89%
MYB	1	107	0.93%
MYB_related	4	112	3.57%
Nin-like	2	20	10.00%
TCP	2	31	6.45%
Trihelix	2	48	4.17%
WRKY	1	90	1.11%

## Conclusions

In this study, we identified 23 *PEBP* family members in quinoa. We investigated the relationships of *PEBP* genes in sugar beet and relatives of quinoa diploid ancestors, and identified 7 orthologous *PEBP* gene pairs. Evolution analysis indicated the reasons for *PEBP* duplication events varied, and tandem duplication is the major driving force for *PEBP* family expansion. Then, we identified the pivotal *PEBP* genes for inflorescence branching, seed germination and flowering time regulation, and predicted 64 putative transcription factors upstream of *CqFTL5* and *CqFTL8* by performing co-expression network analysis.

## Data availability statement

The data presented in the study are deposited in the NCBI SRA repository (http://trace.ncbi.nlm.nih.gov/Traces/sra), accession number PRJNA824606, PRJNA825321, PRJNA824547,PRJNA824641, PRJNA824640, PRJNA824668, PRJNA824959, PRJNA824960, PRJNA824961, PRJNA824962 and PRJNA824963.

## Author contributions

QW conceived and designed this study. QW performed most of the bioinformatics analysis and wrote the manuscript. XB participated in phylogenetic analysis and evolution analysis. MN, LL and YL cultivated the seedlings and harvested the samples for RNA sequencing. YF, CL, XY and LZ participated in expression analysis. All authors contributed to the article and approved the submitted version.

## Supplementary material

Supplementary File 1 Protein sequences of PEBPs from Chenopodium quinoa, C. pallidicaule, C. suecicum, Beta vulgaris, Spinacia oleracea, Arabidopsis thaliana and Oryza saltiva.

Supplementary File 2 Expression data of *PEBP* genes in various biological events.

Supplementary File 3 Expression profiles of the TFs in blue module.
